# Subcritical dimethyl ether extraction as a simple method to extract nutraceuticals from byproducts from rice bran oil manufacture

**DOI:** 10.1038/s41598-020-78011-z

**Published:** 2020-12-03

**Authors:** Donporn Wongwaiwech, Monthana Weerawatanakorn, Panatpong Boonnoun

**Affiliations:** 1 Department of Agro-Industry, Faculty of Agriculture, Natural Resources and Environment, Naresuan University, 99 Moo 9, Tha Pho, Mueang, Phitsanulok, 65000 Thailand; 2 Department of Industrial Engineering, Chemical Engineering Program, Faculty of Engineering, Naresuan University, 99 Moo 9, Tha Pho, Mueang, Phitsanulok, 65000 Thailand

**Keywords:** Biotechnology, Chemistry

## Abstract

The byproducts of rice bran oil processes are a good source of fat-soluble nutraceuticals, including **γ-**oryzanol, phytosterol, and policosanols. This study aimed to investigate the effects of green technology with low pressure as the subcritical fluid extraction with dimethyl ether (SUBFDME) on the amount of γ-oryzanol, phytosterol, and policosanol extracted from the byproducts and to increase the purity of policosanols. The SUBFDME extraction apparatus was operated under pressures below 1 MPa. Compared to the chemical extraction method, SUBFDME gave the highest content of γ-oryzanol at 924.51 mg/100 g from defatted rice bran, followed by 829.88 mg/100 g from the filter cake, while the highest phytosterol content was 367.54 mg/100 g. Transesterification gave the highest extraction yield of 43.71% with the highest policosanol content (30,787 mg/100 g), and the SUBFDME method increased the policosanol level from transesterified rice bran wax to 84,913.14 mg/100 g. The results indicate that the SUBFDME method is a promising tool to extract γ-oryzanol and phytosterol and a simple and effective technique to increase the purity of policosanol. The study presented a novel technique for the potential use of SUBSFDME as an alternative low-pressure and low-temperature technique to extract **γ-**oryzanol and phytosterol. The combination of transesterification and the SUBFDME technique is a potential simple two-step method to extract and purify policosanol, which is beneficial for the manufacture of dietary supplements, functional foods and pharmaceutical products.

## Introduction

A previous study found that coproducts and byproducts from rice bran oil, which are processed using both solvent and cold-pressed extraction systems, such as defatted rice bran, rice bran wax, filter cake, and acid oil, contained high amounts of nutraceuticals, including γ-oryzanol, tocopherol, tocotrienol, phytosterol, and long-chain alcohols, which indicate their commercial potential as a source of functional ingredients^1^. The results offer an idea for investigating the extraction and purification methods of these nutraceuticals from these byproducts. Overwhelming evidence supports the valuable bioactivities of γ-oryzanol, tocotrienol, tocopherol, phytosterol, and policosanol (PCs), including the reduction of oxidative stress activities, anticancer effects, anti-inflammatory properties, cholesterol-lowering effects and protection against cardiovascular disease^2–5^. Of these nutraceuticals, PCs are one of many items of research focusing on their bioactivity related to the decrease in blood cholesterol.

PCs are a mixture of high-molecular-mass primary alcohols ranging in length from 24 to 34 carbon atoms. It is naturally found in wax components, especially rice bran, perilla seed, sugar cane, bee wax, maize, whole grain, etc^5^. Various bioactivities of PCs have been reported, including antioxidant activity, antidiabetic effects, liver protection activity, anti-inflammatory activities and cholesterol-lowering effect^6–9^. Among all of these health benefit properties, the outstanding bioactivity of PCs is the cholesterol-lowering property, which is indicated in 112 articles involving cholesterol-lowering activity published on the PubMed website. The inhibition of hydroxy-methylglutaryl-coenzyme A (HMG-CoA) reductase is the rate-controlling enzyme of the mevalonate pathway, which has been shown to be the cellular mechanism that decreases serum cholesterol levels^5^. Commercially, PCs are available in the market as a dietary supplement that is mostly isolated and purified from various sources such as sugar cane wax (*Saccharum officinarum* L.) or rice bran wax (*Oryza sativa* L.).

In recent years, climate change and interest in environmental preservation have promoted the development of “green technology” for the extraction and isolation of nutraceuticals. In addition, consumers are now worried about organic solvent residues caused by traditional extraction techniques, which hinder further applications of natural functional ingredients. Consequently, many researchers have attempted to develop the process of extraction and purification without the application of hazardous chemicals. Supercritical fluid and subcritical fluid techniques are known as ecofriendly extraction techniques to extract various bioactive compounds that are heat-sensitive, easily oxidized, and decomposed. Supercritical fluid extraction (SUPFE) is broadly used to extract bioactive substances, including oryzanol and phytosterol, from various types of plants, such as Kalahari melon seed oil^10^, roselle seeds^11^, rice bran^12^, buckthorn seeds^13^, soybeans^14^ and melon seeds^15^. However, the SUPFE requires high demands of pressure (100–690 bar), temperature (35–87 °C), energy, and commercial investment, whereas the subcritical fluid extraction technique (SUBFE) requires lower pressure, temperature, energy, and investment, which makes it cheaper and more practicable for large-scale application^16,17^.

SUBFE is a developing technology that uses the properties of extraction solvents, such as the temperature between its boiling point and critical temperature. The solvent is maintained in a liquid state under sufficient pressure^18^. Different solvents have been used in SUBFE, but dimethyl ether (DME) with the formula CH_3_OCH_3_ is one of the “alternative solvents” that receives attention from researchers. The reason is that subcritical fluid extraction with dimethyl ether (SUBFDME) can extract at a low boiling point (− 24.8 °C) with a saturated vapor pressure of 0.51 MPa (20 °C). DME is soluble at low levels in water (7–8 wt%at room temperature), which makes it easy to separate from water and volatile solvents^19^. It has an excellent property for dissolving hydrophobic compounds with no solvent residue in the extracted products. DME has been authorized by the European Food Safety Authority as a safe extraction solvent to produce foodstuffs and food ingredients^20^. Apart from its application as a fuel, subcritical DME has been used for biologically active, flavoring or pungent organic compounds from spices (ginger, black pepper, and chili powder), and its effectiveness is comparable with supercritical carbon dioxide^21^. Most studies on SUPFE have focused on the extraction yield, pressure, temperature, flow rate, and time, but did not focus on the purity of bioactive compounds extracted from SUPFE. These studies highlight that SUBFDME is a promising green technology with a low pressure to increase the purification of bioactive compounds such as policosanol. It is also a promising technology to extract phytosterol and gamma-oryzanol.

According to the available literature, there are no reports on the use of SUBFDME for the extraction of γ-oryzanol, phytosterol, and PCs and for increasing PC purity from the byproducts of rice bran oil processes. The objectives of this study were to investigate the effect of SUBFDME on nutraceutical recovery and the purity of PCs from the byproducts of rice bran oil manufacture. This study focused on the nutraceuticals γ-oryzanol, phytosterol, and PCs.

## Materials and methods

### Materials

The samples from the commercial production of cooking rice bran oil (solvent extraction process), including defatted rice bran (DFRB-S) and rice bran wax (RBW), were provided by Surin Bran Oil Co., Ltd. (Buri Ram, Thailand). Defatted rice bran (DFRB-C) and filter cake (FC) from cold-pressed extraction manufacture as functional rice bran oil were provided by Lopburi Vegetable Oil Industries (Lopburi, Thailand). All defatted rice brans were dried in a hot air oven at 60 °C for 5 h, sieved through a 60-mesh screen and stored at − 20 °C until further analysis. DME was purchased from Siam Tamiya Co., Ltd. (Bangkok, Thailand).

### Standards and reagents

All solvents and chemicals were of analytical grade or HPLC/GC grade and purchased from RCI Labscan (Bangkok, Thailand). The total γ-oryzanol (98.5%) standard was purchased from Tsuno Rice Fine Chemical Co., Ltd. (Wakayama, Japan). PC standards, including docosanol (C_22_), tetracosanol (C_24_), hexacosanol (C_26_), octacosanol (C_28_) and triacontanol (C_30_), and phytosterol standards, including campesterol, stigmasterol, β-sitosterol, sitostanol, and 5α-cholestane, were purchased from Sigma-Aldrich (St. Louis, MO, USA). N,O-bis(trimethylsilyl)-trifluoroacetamide (BSTFA) with 1% trimethylchlorosilane (TMCS) and pyrogallol were also purchased from Sigma-Aldrich (St. Louis, MO, USA).

### Extraction of nutraceuticals from byproducts

The byproducts for the nutraceutical extraction included DFRB-S, RBW, DFRB-C and FC from rice bran oil processes. The extraction method of the SUBFDME technique was used to extract nutraceutical compositions, including γ-oryzanol, phytosterol, and PCs. For RBW, the chemical extraction method of transesterification (TE) was used because it could not be directly used by the SUBFE in the DME method. Releasing DME into the environment decreases the temperature of DME to lower than − 11 °C (data from our experiment), which makes RBW freeze (the wax will solidify at temperatures lower than 25 °C). FC was used as a sample to compare the effect of SUBFDME method with the TE chemical reaction.

### Nutraceutical extraction of byproducts by SUBFDME

**Figure 1:**
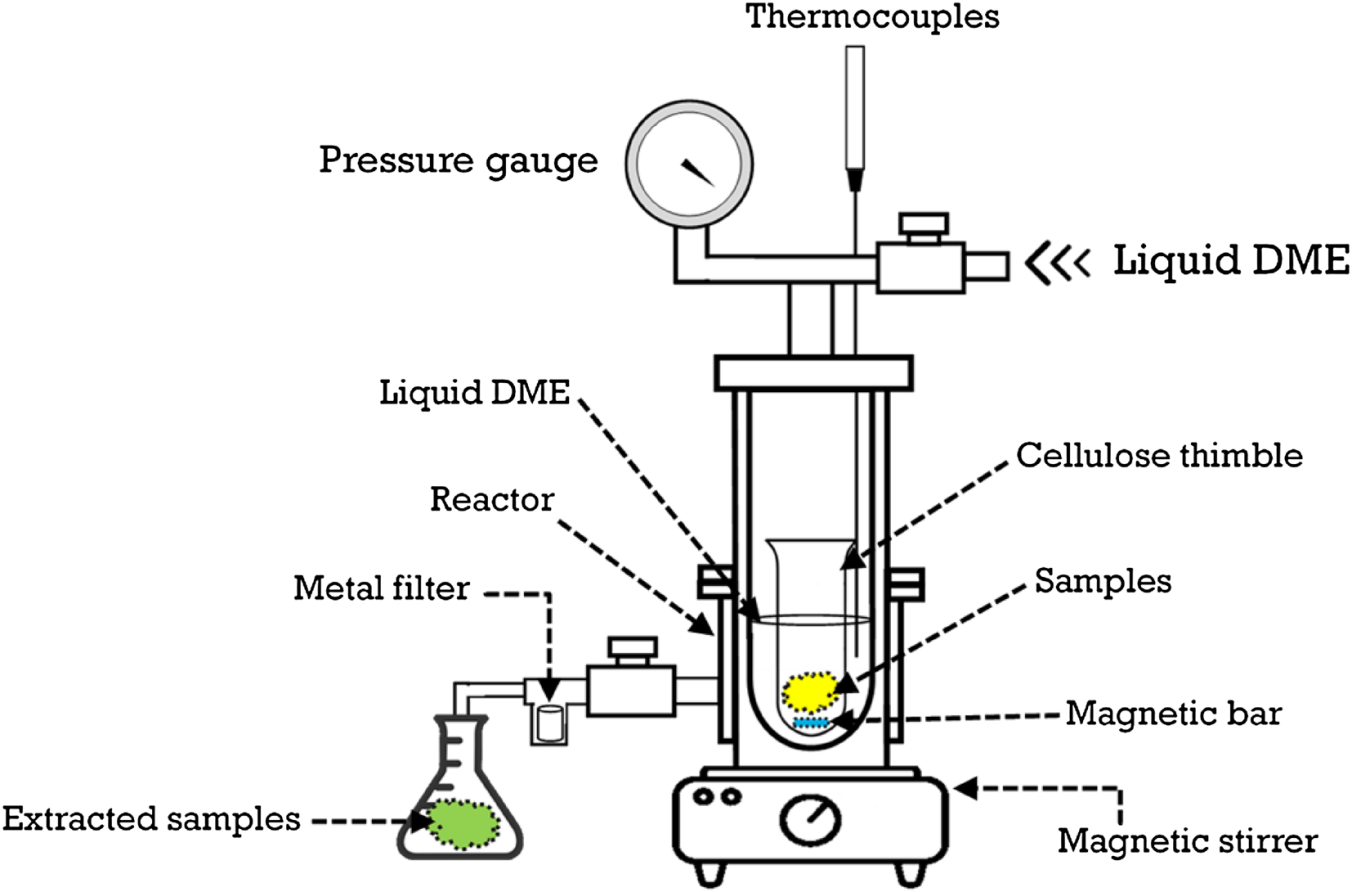
Schematic illustration of the lab-scale SUBFDME apparatus ( adapted from Horikoshi et al. (2008))^36^.

The lab-scale apparatus of SUBFDME with a capacity of 10 g was used to extract nutraceuticals, including γ-oryzanol, phytosterol, and PCs, and a schematic illustration is shown in Fig. 1. The sample was put into a cellulose thimble (Whatman 30 mm × 100 mm), and a known amount of DME was released into the apparatus. The temperature of the reactor, pressure (< 1 MPa), extraction time, and stirring speed rate were set at one condition, and the extraction time was 30 min. The extracted sample was filtered through a metal filter (7 µM) and stored at − 20 °C for further application. The obtained extract from the machine was processed to analyze the bioactive composition.

### Nutraceutical extraction of byproducts by TE

The TE was prepared using a modified method of Aryusuk^22^ by dissolving samples in a solution of NaOH in EtOH (2%) and stirring at 80 °C for 15 min. The solution was allowed to react and mixed with a warm solution of isooctane and EtOH. The isooctane layer was separated and kept in a refrigerator (4 °C) overnight. The crystallized wax that formed was filtered on a Buchner funnel and washed twice with EtOH. The precipitate was kept and dried in a hot air oven at 60 °C for 5 h. The dried extract was ground and stored at − 20 °C for other applications and chemical analysis.

### Increasing the policosanol content from transesterified RBW and FC

RBW and FC were used as samples to increase the PC content (the purity of PCs). Since the results from the first part indicate that TE increased the PC content of the samples, the sample was pretreated by chemical reaction through TE. Then, the SUBFDME extraction was processed for the transesterified samples, and the solvent extraction method (SE) was used to compare the results. The pretreatment conditions of the samples by TE were identical to those mentioned above.

### Increasing purity of policosanols by SUBFDME and by SE

To increase the PC content, the transesterified samples were processed in a SUBFE machine. The condition of the SUBFDME is identical to that for the aforementioned nutraceutical extraction. The nutraceutical contents of the obtained PCs powder from the machine were determined. The SE by toluene was used to compare the result with SUBFE. For SE, transesterified samples were extracted according to the method described by Wongwaiwech et al.^1^ with some modification. The transesterified sample was dissolved in toluene. The mixture was shaken for 30 min and subsequently centrifuged at 4000 rpm at 10 °C for 15 min. The supernatant was collected into a round flask and evaporated until dry. The residues were gathered and stored at − 20 °C for further analysis.

### Analysis of residual solvents in final PCs extracts

The qualitative and quantitative analysis of residual solvents in the obtained PC compounds was modified from the method of Seo and Shin^23^ performed by headspace gas chromatography coupled with mass spectrometry. Briefly, 0.5 g of extracted PC compounds was weighed in a 20-mL headspace vial, and the vial was closed with an aluminum crimp cap equipped with a Teflon-coated butyl rubber septum. The samples were incubated at 70 °C for 30 min and subsequently injected using the automated headspace sampler. The headspace sampler and transfer line were set at 70 °C and 150 °C. The loop equilibrium, loop fill, and GC cycle times were 0.05, 0.10, and 50 min, respectively.

The instrument assembly consisted of an Agilent Technologies 6890 coupled with an Agilent 7694 Headspace-Sampler. The capillary column was an Agilent DB-5 ms (30 m × 0.25 mm × 0.25 µm, USA). Helium was used as a carrier gas at a flow rate of 1.0 mL/min. The pressure exerted by a constant column of 6.75 psi, whereas the sample inlet was held at 250 °C. Oven temperatures originally set at 35 °C (5 min) were subsequently raised to 300 °C (1 min) at a rate of 10 °C/min. The injector, MS quad temperatures, transfer line, and MS source were 250, 150, 280, and 230 °C, respectively. The residual solvent was identified and quantified by the SIM (single ion monitoring) mode according to their retention times and MS spectra. Isooctane, DME, ethanol, toluene, hexane, acetone and acetaldehyde were used as external standards.

### Nutraceutical analyses

#### Analysis of γ -oryzanol contents

γ-Oryzanol was extracted and determined according to a previous report by Wongwaiwech et al.^1^ using LC–MS. Briefly, samples were extracted with a mixture of chloroform and methanol. A solution of 500 µL of supernatant was mixed with a solution of 500 µL of acetonitrile, methanol, and isopropanol, which was subsequently injected into the LC–MS.

γ-Oryzanol was separated on an Agilent Technologies 1100 with a diode array detector (DAD) chromatographic system equipped with an ultraviolet (UV) detector set at 298 and 325 nm. The sample was separated on an Agilent Zorbax Eclipse XDB-C18 column (4.6 m × 150 mm × 5 µm, U.S. A), and the column temperature was set at 40 °C. The mass spectrometer was an Agilent Technologies LC/MSD SL equipped with an electrospray ion source (ESI). The ESI–MS spectra were acquired in the positive ionization mode with a capillary voltage of 4000 V, nebulizer pressure of 50 psi, gas temperature of 350 °C, drying gas of 13.01 L/min and recorded on a mass range of m/z 200–800. A standard mixture of γ-oryzanol was used as an external standard to identify the peaks by Agilent Mass Hunter software based on their retention times.

#### Analysis of phytosterol contents

The phytosterol composition of the samples was analyzed using GC–MS according to a previous method^1^. In summary, the sample was extracted by 60% KOH, 95% ethanol, and 10% NaCl under nitrogen gas (N_2_). The saponified solution was extracted twice with a mixture of hexane and ethyl acetate (9:1, v/v). The unsaponifiable residue was collected and evaporated to dryness. The derivatization was performed by *N*,*O*-bis (trimethylsilyl)-trifluoroacetamide, and the quantification and identification of phytosterol were performed by an Agilent Technologies 7683 on DB-5 ms (30 m × 0.25 mm × 0.25 µm, USA). Trimethylsilyl-phytosterols were identified and quantified in the SIM mode according to their mass spectra and retention times.

#### Analysis of PCs contents

The extraction of PCs was performed according to a previous report by Wongwaiwech et al.^1^. All crude extracts were extracted by the saponification reaction. In brief, a sample was extracted with 0.2 M NaOH (10 mL) in methanol solution and subsequently extracted again with toluene. The upper layer was collected and filtered through a 0.45-µm filter. Identification and quantification of the PCs were determined using an Agilent Technologies 6890 fitted with an Agilent DB-5 ms fused silica capillary column (30 m × 0.25 mm × 0.25 µm, USA). The SIM mode was set for identified and quantified PCs; docosanol (C_22_), tetracosanol (C_24_), hexacosanol (C_26_), octacosanol (C_28_) and triacontanol (C_30_) were identified and quantified according to their molecular target ion and retention times.

### Statistical analysis

Analysis of variance (ANOVA) was performed to analyze the data with Duncan’s tests using the SPSS 19 software (SPSS Inc., Chicago, IL, USA). The significant difference level was set at 0.05. Each reported value is expressed as the mean ± standard deviation (SD) based on the dry weight from three replications.

## Results and discussion

### Extraction yield and nutraceutical contents by SUBFDME and TE

**Figure 2:**
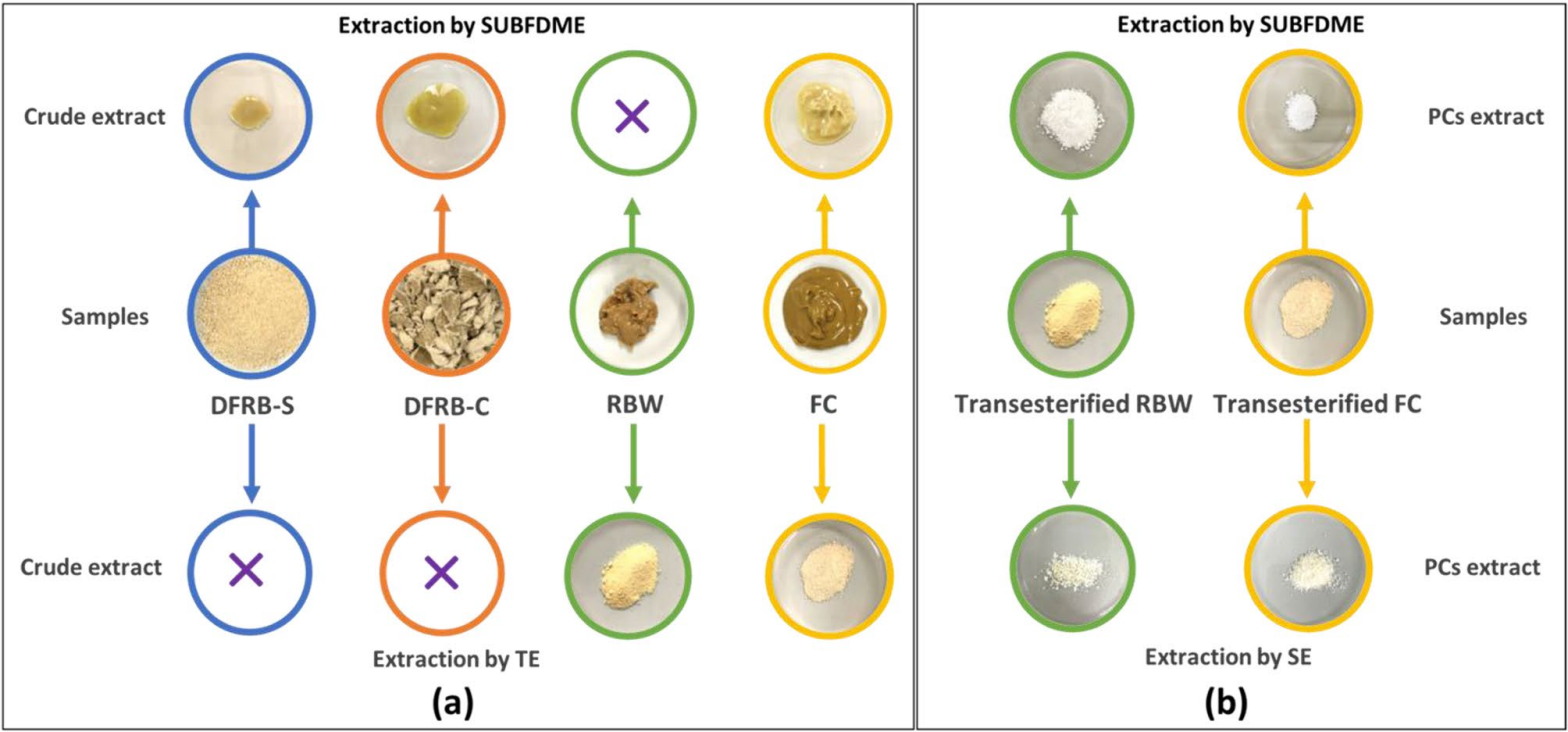
(**a**) Byproducts of rice bran oil refining processes and the resulting crude wax extracted by SUBFDME and TE; (**b**) PCs extract by SUBFDME and SE of transesterified RBW and FC. *DFRB-S* defatted rice bran from the solvent extraction process, *DFRB-C* defatted rice bran from the cold pressed extraction process, *FC* filtered cake, *RBW* rice bran wax, *TE* extracted by transesterification, *SUBFDME* extracted by the subcritical dimethyl ether extraction, *SE* extracted by the solvent extraction.

The contents of γ-oryzanol, phytosterol, and PCs from the crude extract were investigated by the SUBFDME and TE methods. The samples extracted by SUBFDME were a yellowish brown oily substance, while those extracted by the chemical reaction of TE were in a solid form, and their color was similar to their origin samples (Fig. 2a). The extraction yield and conditions for SUBFDME and TE are shown in Table 1, and the nutraceutical contents, including γ-oryzanol, phytosterol and PCs, by SUBFDME and TE are shown in Table 2. For SUBFE, DFRB-C gave a higher extraction yield (9.71%) than DFRB-S (3.60%). From Table 2, our previous study^1^ reported that the DFRB-C byproduct contained more **γ -**oryzanol, phytosterol, and PCs (280.74 mg/100 g) than DFRB-S (77.93 mg/100 g). However, the total amount of nutraceutical compounds found from DFRB-C by SUBFE was slightly lower (1194.96 mg/100 g) than that found from DFRB-S (1233.51 mg/100 g) using the same extraction technique. Since we focused on three bioactive compounds, the higher extraction yield from DFRB-C might contribute to the oil contents and other bioactive components, such as tocopherol, tocotrienal, squalene, phytic acid, lecitin, inositol and wax^3,24^. There are reports of higher amounts of tocopherol tocotrienal, phenolic, phytic acid, inositol and **γ-**oryzanol in defatted rice bran^24,25^. Compared to the report by Wongwaiwech et al.^1^ for defatted rice bran, higher amounts of **γ-**oryzanol and phytosterol were found from crude extracts from SUBFDME. The results suggest a high ability of SUBFDME to release γ-oryzanol (924.51 and 737.46 mg/100 g) and phytosterol (257.12 and 367.54 mg/100 g) from these byproducts (Table 2). For both SUBFDME and TE extraction methods, the most abundant phytosterol contents in the crude extract from all byproducts were stigmasterol and β-sitosterol, and the data were consistent with our previous report^1^. These data were also confirmed by Derakhshan-Honarparvar et al.^26^ and Sawadikiat and Hongsprabhas^27^, which shows that the predominant forms of phytosterol components in rice bran were stigmasterol and β-sitosterol. Regarding FC, SUBFE gave a higher extraction yield (52.14%) and bioactive compounds, including **γ-**oryzanol (829.88 mg/100 g) and phytosterol (312.34 mg/100 g), than TE (18.82%). There was a slightly lower oryzanol content (829.88 mg/100 g) of crude extract from the SUBFDME than the report of oryzanol content in FC (1058.28 mg/100 g) by Wongwaiwech et al.^1^. This is due to the analysis of **γ-**oryzanol by Wongwaiwech et al.^1^, who used the solvent extraction method, which is well known for its high ability to extract bioactive compounds^28^.

**Table 1:** Comparison of the extraction yield of nutraceutical from byproducts using the SUBFDME and TE processes.

Samples	Methods	Solvent	Extraction times	Extraction yields (%)
DFRB-S	SUBFDME	liquified dimethyl ether	30 min	3.60 ± 1.34
DFRB-C	SUBFDME	liquified dimethyl ether	30 min	9.71 ± 3.03
FC	SUBFDME	liquified dimethyl ether	30 min	52.14 ± 3.62
FC	TE	Ethanol + Isooctane	20 h	18.82 ± 5.10
RBW	TE	Ethanol + Isooctane	20 h	43.71 ± 8.64

*DFRB-S* defatted rice bran from the solvent extraction system, *DFRB-C* defatted rice bran from the cold-pressed extraction system, *FC* filtered cake from the cold-pressed extraction system, *RBW* rice barn wax from the solvent extraction system, *SUBFDME* extracted by the subcritical dimethyl ether extraction, *TE* tranesterification.

**Table 2:** Comparison of γ-oryzanol, phytosterol and policosanol contents extracted from byproducts using various extraction methods.

Nutraceuticals (mg/100 g sample)	Wongwaiwech et al., 2019				Extraction by SUBFDME			Extraction by TE	
	Samples				Samples			Samples	
	DFRB-S	DFRB-C	FC	RBW	DFRB-S	DFRB-C	FC	FC	RBW
γ-Oryzanol	39.39 ± 0.16	229.76 ± 1.52	1058.28 ± 24.86	862.80 ± 5.53	924.51 ± 3.80^a^	737.46 ± 9.14^c^	829.88 ± 18.66^b^	85.54 ± 3.14^d^	43.16 ± 1.42^e^
**Phytosterol**									
Campesterol	0.34 ± 0.01	0.47 ± 0.14	52.39 ± 2.70	0.74 ± 0.05	63.33 ± 1.12^a^	47.37 ± 0.45^b^	41.28 ± 0.86^c^	12.73 ± 0.23^d^	7.69 ± 0.28^e^
Stigmasterol	0.74 ± 0.01	2.61 ± 0.03	60.41 ± 2.80	21.78 ± 0.86	105.25 ± 1.32^a^	68.85 ± 0.99^b^	53.19 ± 2.70^c^	11.40 ± 0.18^d^	4.62 ± 0.12^e^
β-Sitosterol	0.38 ± 0.01	1.64 ± 0.54	118.03 ± 6.39	66.83 ± 1.44	67.56 ± 4.37^c^	220.64 ± 11.48^a^	204.06 ± 5.47^b^	62.30 ± 3.26^c^	43.48 ± 2.04^d^
Sitostanol	0.27 ± 0.01	0.69 ± 0.20	13.16 ± 1.36	3.86 ± 0.77	20.97 ± 2.23^b^	30.68 ± 1.13^a^	13.81 ± 0.64^c^	6.85 ± 0.78^d^	3.92 ± 0.24^d^
Total phytosterol	1.75 ± 0.01	5.43 ± 0.86	243.99 ± 13.25	93.21 ± 1.39	257.12 ± 0.30^c^	367.54 ± 11.79^a^	312.34 ± 9.66^b^	93.28 ± 2.53^d^	59.71 ± 2.11^e^
**Policosanol**									
C22	3.35 ± 0.01	5.08 ± 0.02	3.75 ± 0.12	7.99 ± 0.22	4.93 ± 0.04^c^	4.02 ± 0.06^c^	3.53 ± 0.05^c^	35.28 ± 0.25^b^	108.35 ± 2.04^a^
C24	13.94 ± 0.94	21.56 ± 0.55	24.25 ± 2.03	53.80 ± 0.11	19.72 ± 0.87^c^	18.33 ± 1.54^c^	20.40 ± 0.86^c^	506.10 ± 13.39^b^	4392.24 ± 61.81^a^
C26	1.72 ± 0.50	1.83 ± 0.03	11.57 ± 0.17	42.75 ± 1.95	7.73 ± 0.12^c^	7.93 ± 0.13^c^	7.33 ± 0.04^c^	730.28 ± 16.45^b^	4103.22 ± 24.74^a^
C28	11.12 ± 0.31	10.15 ± 0.45	12.13 ± 0.27	71.84 ± 2.66	9.38 ± 0.29^c^	10.02 ± 0.38^c^	13.39 ± 0.28^c^	2088.31 ± 26.15^b^	10404.91 ± 56.85^a^
C30	6.66 ± 0.13	6.91 ± 0.17	24.30 ± 0.71	156.41 ± 2.9	10.13 ± 0.50^c^	9.67 ± 0.47^c^	13.84 ± 0.26^c^	2740.14 ± 21.33^b^	11,779.16 ± 15.09^a^
Total policosanol	36.79 ± 0.48	45.55 ± 0.14	76.01 ± 3.29	332.79 ± 7.28	51.88 ± 1.82^c^	49.96 ± 2.59^c^	58.48 ± 0.35^c^	6100.12 ± 77.57^b^	30,787.89 ± 130.35^a^

Each value represents the mean ± S.D. Values with different superscript letters in the same row are significantly different (P < 0.05). Values in the table are expressed on a dry basis. The γ-oryzanol, phytosterol and policosanol contents of the original samples were extracted by the solvent extraction method (data from our previous study^1^.

*TE* extracted by transesterification, *SUBFDME* extracted by the subcritical dimethyl ether extraction, *DFRB-S* defatted rice bran from the solvent extraction system, *DFRB-C* defatted rice bran from the cold pressed extraction system, *RBW* rice bran wax from the solvent extraction system, *FC* filtered cake from the cold pressed extraction system.

Compared to SUBFDME, the TE method for FC gave dramatically higher PC contents (6100.12 mg/100 g). There was no significant difference in PC contents of the crude extract from all byproducts by SUBFDME; therefore, SUBFDME had no effect on PC contents regardless of the type of byproduct. Wongwaiwech et al.^1^ reported a high amount of PCs in RBW (332.79 mg/100 g). Many ^1^reports indicate that RBW is a good source of PC compound^29^. Interestingly, the TE method released very high amounts of PCs from RBW at 30,787.89 mg/100 g (Fig. 2a), which is nearly 93 times the amount previously reported by Wongwaiwech et al.^1^. The chemical reaction of TE is very efficient as an extraction method for PCs from RBW^30,31^. Wang et al.^32^ and Ning-ning et al.^33^ used the transesterification method to extract policosanol from rice bran wax and found that the yield of policosanol was approximately 21%. Here, we found a lower yield of PC (13.46%) by TE extraction compared to Wang et al. and Ning-ning et al.’s report (21%). Different refining processes of rice bran oil manufacture and cultivar variations of rice bran affected the yield of PCs.

The results indicate that a powerful extraction technique for **γ-**oryzanol and phytosterol is the SUBFDME method, whereas the TE method is effective in PC extraction. Since DME can dissolve a wide range of nonpolar substances^34^, it can increase mass transfer by developing hydrogen bonds with extractable substances^35^. The principle of subcritical fluid extraction promotes the DME temperature to rise above the boiling point and applies sufficient pressure to help maintain the DME in a liquid state. Under such conditions, DME has features that help promote the extraction process, such as high diffusion coefficients, low viscosity and high solvent strength. Furthermore, an increased temperature produces high solubility and high diffusion rates of the solutes in the solvent, while pressure helps to force DME into the sample matrix and allows it to be filled faster^36^. The data also suggest that the SUBFDME technique can liberate fat soluble bioactive compounds, including γ-oryzanol and phytosterol, and it liberates the same amount of PCs regardless of the types of sample tested.

### Increasing PC recovery of transesterified rice bran wax and filter cake by SUBFDME

Both SE and SUBFDME methods can be used to increase the PC content of transesterified FC and RBW; therefore, they were compared in terms of PC content and purity. From the first part of the results, the data suggest that the TE extraction technique released a large amount of PC compounds from both types of byproducts, FC and RBW. FC and RBW were promising sources of PCs and selected as samples to process to increase the PC content. The extraction yields of crude PCs extract from transesterified FC and RBW are shown in Table 3. The extraction yields of crude PCs from transesterified FC and RBW by the SE method (10.84–18.24%) were higher than those from SUBFDME (1.32–2.49%). The color of crude PCs extracted by the SUBFDME and SE methods changed from a yellowish-brown powder to pure white and yellowish-white powder, respectively (Fig. 2b). The color of the extracted PCs changed through a multistep process. Liu et al.^37^ reported that crude PC extracted from RBW appeared dark brown, but after the purification step, the color of the obtained PCs changed to white powder. A comparison of γ-oryzanol, phytosterol and PC contents of extracts from transesterified FC and RBW using the SUBFDME and SE methods is shown in Table 4. The results show that a high amount of γ-oryzanol was detected in the extract from transesterified FC (258.02 mg/100 g), followed by transesterified RBW (114.37 mg/100 g) by the SE method, whereas it was not detected by SUBFDME. These data suggest that SE, not SUBFDME, increases the γ-oryzanol content of the extract from transesterified FC and RBW samples by 3 and 2.6 times, respectively (Table 2). The phytosterol contents of transesterified FC and RBW were 93.28 and 59.71 mg/100 g, respectively (Table 2). The SE method increased the phytosterol content of the extract of transesterified FC and RBW by 6- and 7.7-fold, respectively. Although SUBFDME increased the phytosterol contents of transesterified samples, the increase in phytosterol by SE was more effective than that by SUBFDME. However, the results from Table 2 indicate that direct extraction of byproducts by SUBFDME was the most effective method for both oryzanol and phytosterol, since there was a higher yield with a shorter extraction time than chemical extraction. Chotimarkorn et al.^38^, Lilitchan et al.^39^ and Lai et al.^40^ applied a solvent (methanol) extraction method to extract gamma-oryzanol from Thai rice bran and Japonica rice bran. They reported that the yields of oryzanol were 56.0–108.0 mg/100 g, 343–367 mg/100 g and 160–180 mg/100 g, respectively. Bhatnagar et al.^41^ used Soxhlet extraction to extract phytosterols from broken rice and rice germ, and the yields of phytosterols were 12.87 and 76.96 mg/100 g, respectively. Compared with solvent extraction^38–41^, this study confirmed that SUFDME was significantly more effective for gamma-oryzanol and phytosterol extraction from byproducts (DFRB-S (924.51 and 257.12 mg/100 g, respectively), DFRB-C (737.46 and 367.54 mg/100, respectively), and FC (829.88 and 312.34 mg/100 g, respectively).

**Table 3:** Comparison of extraction yields from transesterified RBW and FC using the SUBFDME and SE methods.

Transesterified samples	Methods	Solvent	Extraction time	Extraction yield (%)
TE-RBW	SUBFDME	Liquified dimethyl ether	30 min	2.49 ± 0.49
TE-FC	SUBFDME	Liquified dimethyl ether	30 min	1.32 ± 0.27
TE-FC	SE	Toluene	3 h	10.84 ± 0.23
TE-RBW	SE	Toluene	3 h	18.24 ± 0.56

*TE-samples* tranesterified samples, *RBW* rice barn wax from the solvent extraction system, *FC* filtered cake from the cold pressed extraction, *SE* extracted by the solvent extraction (toluene) , *SUBFDME* extracted by the subcritical fluid dimethyl ether extraction.

**Table 4:** Comparative contents of gamma-oryzanol, phytosterol and policosanol from tranesterified RBW and FC using the SUBFDME and SE techniques.

Nutraceutical (mg/100 g sample)	Extraction by SUBFDME		Extraction by SE	
	Transesterified samples		Transesterified samples	
	TE-FC	TE-RBW	TE-FC	TE-RBW
γ-Oryzanol	ND	ND	258.02 ± 0.01^a^	114.37 ± 1.18^b^
**Phytosterol**				
Campesterol	ND	ND	201.81 ± 8.59^a^	165.16 ± 1.11^b^
Stigmasterol	ND	19.19 ± 0.24^c^	194.99 ± 6.03^a^	134.22 ± 2.46^b^
β-Sitosterol	32.12 ± 7.92^c^	18.23 ± 2.06^d^	47.78 ± 1.23^b^	90.65 ± 3.47^a^
Sitostanol	161.04 ± 2.02^b^	251.72 ± 7.75^a^	120.13 ± 15.04^c^	71.97 ± 0.11^d^
Total phytosterol	193.17 ± 5.90^d^	289.14 ± 9.87^c^	563.47 ± 28.36^a^	460.16 ± 2.23^b^
**Policosanol**				
C22	203.92 ± 0.73^d^	250.08 ± 1.53^c^	293.04 ± 3.06^b^	374.82 ± 2.62^a^
C24	10,318.48 ± 511.04^c^	13,374.37 ± 9.35^a^	12,231.18 ± 63.84^b^	12,483.98 ± 302.26^b^
C26	14,153.56 ± 406.18^a^	14,613.22 ± 291.18^a^	12,556.02 ± 139.01^b^	10,711.60 ± 527.84^c^
C28	32,560.01 ± 389.59^a^	31,898.52 ± 742.87^a^	24,164.37 ± 266.73^b^	21,056.39 ± 208.28^c^
C30	27,162.89 ± 135.30^a^	24,776.45 ± 160.15^b^	23,073.60 ± 252.44^c^	18,090.92 ± 109.98^d^
Total PC	84,398.86 ± 1,362.92^a^	84,913.14 ± 1,409.00^a^	72,318.21 ± 725.02^b^	62,717.72 ± 546.46^c^

Each value represents the mean ± SD. Values with different superscript letters in the same row are significantly different (P < 0.05). Values in the table are expressed on a dry basis.

*ND* amount detected below the LOD; the LOD of gamma-oryzanol is 0.05 ppm, *TE-samples* tranesterified samples, *SUBFDME* extracted by the subcritical fluid dimethyl ether extraction, *SE* extracted by solvent extraction (toluene) , *RBW* rice bran wax from the solvent extraction system, *FC* filtered cake from the cold-pressed extraction system.

The PC content was significantly different from the **γ-**oryzanol and phytosterol contents. The SE method increased the PC content of transesterified FC and RBW to 72,318.21 and 62,717.72 mg/100 g, respectively, while SUBFDME was more effective in increasing the PC content of transesterified samples (84,398.86 and 84,913.14 mg/100 g, respectively). The distinctly increasing amounts were increased by 14 and 3 times. This result suggests that FC and RBW are good sources of PCs, and the combination methods of TE and SUBFDME are promising methods to extract and purify PCs from FC and RBW, which are coproducts from the rice bran oil process. Many studies focused on extraction and purification methods for policosanol, such as solvent extraction, supercritical fluid extraction, ultrasonic-assisted extraction, and other extraction methods. Chen et al., 2007^42^ reported that the extraction of rice bran wax by transesterification and purification by molecular distillation made the yield of policosanol 53.8%. Lorenz et al.^43^ used the supercritical carbon dioxide extraction method to isolate policosanol from quine wax and achieved a PC content of 36,410 mg/100 g. Ishaka and colleagues^29^ used solvent ultrasonic-assisted extraction to extract PCs from rice bran wax and rice bran oil, and the yields of policosanol were 10,820 and 9810 mg/100 g, respectively.

This study is the first report using SUBFDME to increase the purity (approximately 84%) of PC compounds. The data also indicate that the remaining (16%) PC compounds may be dotriacontanol (C_32_H_66_O), tetratriacontanol (C_34_H_70_O), phytosterol and vit E (data not shown).

### Residual levels of solvents in PC extracts

**Figure 3:**
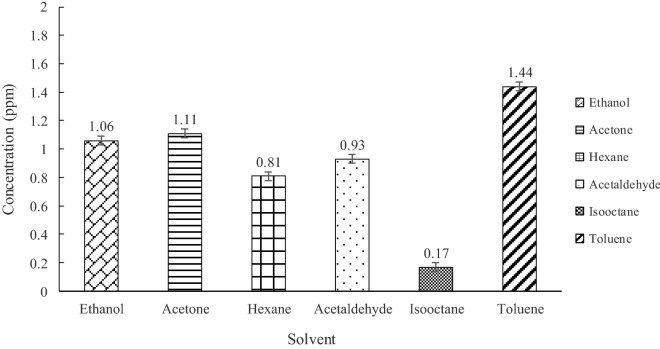
Concentrations of residual solvents from policosanol extracts analyzed by GC/MS.

Since TE was used to extract PCs in this study, the residual solvent in the final PC extract was monitored. The analysis result are shown in Fig. 3. All residual solvents in the TE process were less than 1.50 ppm. Toluene residue (1.44 ppm) was the most frequently detected solvent in the PC extracts following acetone (1.11 ppm) and ethanol residue (1.06 ppm), whereas hexane, acetaldehyde and 2,2,4-trimethylpentane (isooctane) were less than 1 ppm.

The International Council for Harmonization of Technical Requirements for Pharmaceuticals for Human Use (ICH)^44^ has reported recommendations for residual solvent levels considering safety in pharmaceutical products. According to the guidelines, the levels of hexane and toluene are limited to below 290 and 890 ppm, respectively, and the levels of ethanol and acetone should be less than 5000 ppm. However, regarding the guidelines, there are no toxicological data of isooctane on the permitted daily exposure, and Patnaik^45^ reported that the acute toxicity of isooctane was very low and similar to that of *n*-octane. An acceptable intake for the acetaldehyde set in the Food Safety Commission of Japan’s specification^46^ was 1.8 mg/person/day. In conclusion, the obtained PC extract contained negligible amount of residual solvent and all residual levels of solvents in the final PC extracts do not exceed the recommendations.

## Conclusions

The green technology with low pressure and temperature, called SUBFDME, is a promising novel technology to extract **γ-**oryzanol and phytosterol, as it significantly improves their contents. SUBFDME can be directly used to extract policosanol regardless of the type of samples. The simple two-step TE and SUBFDME techniques can be successfully used as extraction and purification models to increase the PC recovery from filter cake and rice bran wax, the low prize of coproducts from the rice bran oil process. SUBFDME has higher efficiency in purified PCs than traditional solvent purification and is an innovative alternative extraction technology to extract fat soluble bioactive compounds, including **γ-**oryzanol phytosterol.

### Ethics statement

The research did not include human subjects or animal experiments.

## Abbreviations

PCs: Policosanols
SUBFDME: Subcritical fluid extraction with dimethyl ether
SUBFE: Subcritical fluid extraction technique
HMG-CoA: Hydroxy-methylglutaryl-coenzyme A
SUPFE: Supercritical fluid extraction
DFRB-S: Defatted rice bran from solvent extraction process of rice bran oil
RBW: Rice bran wax
DFRB-C: Defatted rice bran from cold pressed extraction process of rice bran oil
FC: Filter cake
DME: Liquefied dimethyl ether
TE: Transesterification method
SE: Solvent extraction method
